# Musical Instrument Practice Predicts White Matter Microstructure and Cognitive Abilities in Childhood

**DOI:** 10.3389/fpsyg.2019.01198

**Published:** 2019-05-24

**Authors:** Psyche Loui, Lauren B. Raine, Laura Chaddock-Heyman, Arthur F. Kramer, Charles H. Hillman

**Affiliations:** ^1^ Department of Music, Department of Psychology, Northeastern University, Boston, MA, United States; ^2^ Beckman Institute, University of Illinois, Champaign, IL, United States

**Keywords:** music, language, cognition, neuroimaging, brain structure, intelligence

## Abstract

Musical training has been associated with advantages in cognitive measures of IQ and verbal ability, as well as neural measures including white matter microstructural properties in the corpus callosum (CC) and the superior longitudinal fasciculus (SLF). We hypothesized that children who have musical training will have different microstructural properties in the SLF and CC. One hundred children aged 7.9–9.9 years (mean age 8.7) were surveyed for their musical activities, completed neuropsychological testing for general cognitive abilities, and underwent diffusion tensor imaging (DTI) as part of a larger study. Children who play a musical instrument for more than 0.5 h per week (*n* = 34) had higher scores on verbal ability and intellectual ability (standardized scores from the Woodcock-Johnson Tests of Cognitive Abilities), as well as higher axial diffusivity (AD) in the left SLF than those who did not play a musical instrument (*n* = 66). Furthermore, the intensity of musical practice, quantified as the number of hours of music practice per week, was correlated with axial diffusivity (AD) in the left SLF. Results are not explained by age, sex, socio-economic status, or physical fitness of the participants. The results suggest that the relationship between musical practice and intellectual ability is related to the maturation of white matter pathways in the auditory-motor system. The findings suggest that musical training may be a means of improving cognitive and brain health during development.

## Introduction

The impact of music training on human brain and cognitive development has been a topic of intense interest in recent years (Kraus and Chandrasekaran, 2010). Music perception skills in children are correlated with performance on phonological awareness and reading tests (Lamb and Gregory, 1993; Anvari et al., 2002), as well as on general performance on IQ tests (Lynn et al., 1989). Children who take music lessons outperform their musically untrained counterparts in tests of verbal memory and reading ability (Hurwitz et al., 1975; Ho et al., 2003). Children who initially perform below the mean in academic achievement tests, after a year of musical training, catch up with their musically untrained counterparts in academic achievement (Gardiner et al., 1996). While these results suggest a relationship between music training and cognitive abilities, the direction of causality is unclear.

Randomized controlled trials provide a stronger test of the direction of causality, but their results are more mixed. Positive evidence comes from a randomized controlled trial in 144 six-year-olds, comparing 36 weeks of lessons in keyboard, voice, and drama against no-training controls, which found that children in the music groups exhibited greater increases in full-scale IQ than no-training controls (Schellenberg, 2004). Another randomized controlled trial comparing children in music and visual art training showed that children in the music group performed better on verbal intelligence measures after only 20 days of training, compared to no changes in the visual art group (Moreno et al., 2011). Furthermore, children in a school-based music program with weekly 45-min instrumental lessons showed greater improvements in verbal memory than children in science-education and no-training control groups, even after controlling for socio-economic status (SES), age, and IQ; in contrast, no differences between groups were found in the visual memory tests (Roden et al., 2012). Another longitudinal randomized controlled trial showed that children who underwent instrumental music training outperformed visual art training and no-intervention controls on neuropsychological tasks of inhibition, planning, and verbal IQ (Jaschke et al., 2018). In a further mediation analysis, the authors showed that performance on these neuropsychological tasks explained music training-related increases in standardized academic achievement scores, suggesting a far transfer effect from music education to academic achievement mediated by executive functions (Jaschke et al., 2018).

On the other hand, there are also training studies that have not observed transfer effects from musical training to non-musical cognitive tasks including receptive vocabulary, numerical discrimination, visual form analysis, and spatial navigation (Mehr et al., 2013). A meta-analysis of the effects of music training on children’s cognitive and academic skills showed small effect sizes in transfer to intelligence, memory, mathematics, phonological processing, and spatial processing, with effect sizes being affected by methodological considerations: smaller effect sizes were generally observed in studies with randomized designs and in studies that compared music training against active control groups (Sala and Gobet, 2017). However, this meta-analysis did not take into account the intensity of musical training, which strongly influences the relationship between music training and IQ (Schellenberg, 2006). A fuller understanding of the relationship between music training and cognition may come from including information about the intensity of musical practice, as well as characterization of the underlying neural mechanisms.

Functional neuroimaging studies have examined the mechanisms underlying musical training effects on verbal and executive function tests in children. Musically trained children perform better on behavioral measures of verbal fluency using timed neuropsychological tests and fMRI measures during executive function tests such as task switching and rule representation (Zuk et al., 2014). Children with musical training and children with physical activity training both showed a stronger Stroop effect than no-training controls, coupled with more activity in the inferior frontal gyrus, supplementary motor area, and anterior cingulate cortex in the music group compared to the no-treatment group, with the physical activity group falling in between the music and no-treatment groups (Sachs et al., 2017). The results suggest that music training may transfer to executive functions by acting on the auditory-motor and cognitive control networks in the brain.

Longitudinal as well as cross-sectional studies have identified a network of areas associated with music training in auditory-motor regions that are shared with other functions such as language and dance (Sluming et al., 2002; Bermudez et al., 2009; Hyde et al., 2009; Karpati et al., 2017). A longitudinal study found that cumulative hours of music practice in children and adults was correlated with functional activation in the left supramarginal gyrus, part of the auditory-motor network, during music listening (Ellis et al., 2013). Early onset of musical training in childhood is associated with larger gray matter volume and higher cortical surface area in auditory-motor areas including the superior temporal lobe and inferior frontal lobe (Bailey et al., 2014) as well as increased fractional anisotropy (FA) in the temporal lobe and the corpus callosum (Steele et al., 2013).

A prominent white matter pathway that connects the temporal lobe and the frontal lobe is the superior longitudinal fasciculus (SLF), which includes the arcuate fasciculus. Microstructural properties of the SLF are associated with reading skills in children (Yeatman et al., 2011; Saygin et al., 2013). Fractional Anisotropy arcuate fasciculus is higher among people who excel at learning languages and grammatical structures (Floel et al., 2009; Qi et al., 2015) as well as new musical structures (Loui et al., 2011; Vaquero et al., 2018). People with congenital amusia, who have difficulty perceiving and producing pitch and melody, show reduced connectivity in the arcuate fasciculus (Loui et al., 2009), and diffusion properties in the arcuate and other frontal white matter pathways predict recovery from acquired amusia for stroke patients (Sihvonen et al., 2016, 2017). People with music training have shown both increases and decreases in FA and volume in the SLF (Oechslin et al., 2010; Halwani et al., 2011), as supported by cross-sectional comparisons and randomized training studies (Moore et al., 2017). FA of the SLF is also related to language ability and exposure (Romeo et al., 2018), as well as to age (Lebel et al., 2008; Krogsrud et al., 2016) and socio-economic status (Gullick et al., 2016).

In addition to the SLF, the corpus callosum (CC) is a white matter pathway that has shown differences as a result of early musical training. The anterior half of the CC is larger in musically trained adults, especially in those who started musical training before the age of 7 (Schlaug et al., 1995). The age of onset of musical training is correlated with microstructural properties of the CC, with musicians who began training earlier showing higher FA and lower radial diffusivity (RD) especially in the midpoint of the CC (Steele et al., 2013). More specific evidence for the effect of early musical training comes from a longitudinal study comparing children after 2 years of training in music, in physical activity, and in a no-training control group, which showed highest FA in the CC of the music group, specifically in the crossing pathways connecting superior frontal, sensory, and motor segments (Habibi et al., 2017).

Taken together, mounting evidence suggests that musical training affects verbal ability, with more limited effects on general intellectual ability, and the underlying neural substrates most likely involve connectivity between frontal and temporal lobe regions and between left and right midline hemispheres, with white matter effects centering around the SLF and the CC.

Studies reviewed thus far have controlled for multiple possible sources of confounds in assessing the effects of musical training on brain and cognitive development. All of the studies reviewed herein controlled for age, sex, and socioeconomic status, and most also controlled or specifically examined the duration, intensity, and/or age of onset of musical training. Interestingly, studies on effects of musical training thus far have not controlled for physical activity. The effects of physical activity on brain and cognitive function have become increasingly clear in recent years (Hillman et al., 2008; Khan and Hillman, 2014). Aerobic fitness is specifically related to executive control, with more fit individuals showing stronger neural and cognitive indices of attention and executive function (Kramer et al., 1999; Donnelly et al., 2016). Since music making is a mild form of physical activity, persistent musical practice may require or enhance aerobic fitness, thus moderating the relationship between music training and brain and cognitive measures. Here, we assess the relationship between musical training and standardized measures of verbal ability and general intellectual ability and relate these to diffusion measures of the SLF and CC in a large sample of children, while controlling for possible sources of variability from aerobic fitness as well as age, sex, and socioeconomic status.

## Materials and Methods

### Subjects

All behavioral and neuroimaging data from the present sample were collected as part of a larger study on the effects of physical activity on children’s cognitive performance and brain structure and function (Chaddock-Heyman et al., 2014, 2018; Hillman et al., 2014). One hundred children aged 7.9–9.9 provided informed assent as approved by the Institutional Review Board (IRB) of the University of Illinois at Urbana-Champaign (UIUC), and their legal guardians provided written informed consent in accordance with the IRB of UIUC. Children were tested in general reading achievement and neuropsychological tests of cognitive ability. The legal guardians also answered simple questions about their musical experience and training, as detailed below.

### Stimuli and Procedures

#### Musical Experience Measures

Musical experience was assessed by a questionnaire to the parents. Questions included:

1. Does your child participate in musical activities? (Yes vs. No)
2. If yes: Does your child play an instrument? (Yes vs. No)
3. If so, what instrument(s)?
4. Does your child participate in choir? (Yes vs. No)
5. How many hours a week does your child spend participating in musical activities? (Numerical response)

#### Verbal and Intellectual Ability

Intellectual abilities were assessed using the Woodcock-Johnson Tests of Cognitive Abilities (Woodcock et al., 2001). These included measures of Brief Intellectual Ability (BIA standard score) and Verbal Ability (standard score) (Woodcock et al., 2001; Schrank, 2011). Participants completed the Woodcock-Johnson III (WJ III) to assess a range of cognitive abilities. Administration of the WJ III was conducted individually by trained researchers. Various subtests of the WJ III were completed to assess cognitive abilities, and BIA was used to screen for below normal intelligence. A combination of individual subtests was completed to form the Verbal Ability cluster, which can be used for interpretive purposes. The Verbal Ability cluster was computed using the manufacturer’s software, which provides measures of standard scores and percentiles. The WJ III is based on a standard score with a mean of 100 and a standard deviation of 15.

#### Age and Socio-Economic Status

Age was recorded as years on the date of participation. SES was scored as three categories: participants received a “1” if they receive a free or reduced lunch^1^, if both parents have less than a high school education, or if they live in a one-parent household *and* that parent has less than a high school education. Participants received a “3” if one or both parents work *and* have a college education. All other participants received a “2.” Table 1 shows demographics of participants with and without musical instrument training.

**Table 1:** Demographic variables comparing participants with and without musical instrument training.

	Musical instrument training		
	No	Yes	
	Mean (SD) (range)	Mean (SD) (range)	
*N*	66	34	
Age (years)	8.677 (0.5534) (7.9–9.9)	8.733 (0.54) (7.9–9.8)	*t* = 0.49, *p* = 0.63
Sex (*n*)	33°F 33 M	21°F 13 M	*X* ^2^ = 1.25, *p* = 0.26
SES (score)	1.92 (0.81) (1–3)	2.15 (0.66) (1–3)	*t* = 1.48, *p* = 0.14
Music practice intensity (h/week)	0.738 (1.33) (0–5)	2.111 (1.19) (0.5–6)	*t* = 4.48, *p* < 0.01*
WJ III Brief Intellectual Ability (standard score)	107.7 (12.3) (79–132)	117.35 (9.24) (89–133)	*t* = 4.4, *p* < 0.01*
WJ III Verbal Ability (standard score)	107.68 (11.31) (74–132)	116.24 (10.6) (85–151)	*t* = 2.97, *p* = 0.004*
VO_2_max (ml/kg/min)	41.9 (7.14) (26.9–57.9)	44.47 (9.22) (24.4–61.6)	*t* = −1.40, *p* = 0.17

*p < 0.05.

#### Aerobic Fitness Testing

As the present data were collected as part of a larger study on the effects of physical activity training, children also completed a test of cardiorespiratory fitness as described in Chaddock-Heyman et al. (2018). Cardiorespiratory fitness was measured as maximal oxygen consumption (VO_2_max) during a graded exercise test, which employed a modified Balke protocol and was administered on a motor-driven treadmill (LifeFitness, Schiller Park, IL). Expired gases were analyzed using a TrueOne2400 Metabolic Measurement System (ParvoMedics, Sandy, Utah). Children walked and/or ran on the treadmill at a constant speed, with increasing grade increments of 2.5% every 2 min, until volitional exhaustion. Oxygen consumption was measured using a computerized indirect calorimetry system (ParvoMedics True Max 2,400), and averages for VO_2_ and respiratory exchange ratio (RER) were assessed every 20 s. Heart rate (HR) was measured using a polar HR monitor (Polar WearLink+31; Polar Electro, Finland) throughout the test, and ratings of perceived exertion (RPE) were assessed every 2 min using the children’s OMNI scale (Utter et al., 2002). Maximal oxygen consumption was expressed in ml/kg/min, and VO_2_max was based upon maximal effort which was evidenced by four criteria: (1) a plateau in oxygen consumption with an increase of <2 ml/kg/min despite an increase in workload, (2) a peak HR ≥ 185 beats per minute and a plateau in HR (Freedson and Goodman, 1993), (3) RER ≥ 1.0 (Bar-Or, 1983), and/or (4) a rating of ≥8 on the children’s OMNI scale of perceived exertion (Utter et al., 2002). Greater VO_2_max reflects superior cardiovascular fitness.

### Magnetic Resonance Imaging Acquisition

Diffusion-weighted images were acquired on a Siemens Magnetom Trio Allegra 3 T whole-body MRI scanner with a 12-channel receiver head coil, with repetition time (TR) = 4.8 s, echo time (TE) = 100.4 ms, and 3.44 mm^2^ in-plane resolution with 4 mm slice thickness. Thirty-two slices were collected parallel to the anterior-posterior commissure plane to obtain whole-head coverage with no gap. One 30-direction diffusion-weighted echo planar imaging scan (*b* = 1,000 s/mm^2^) and four T2-weighted b0 images (*b* = 0 s/mm^2^) were collected.

### Diffusion Data Analysis

Image analyses were performed using FSL 5.0.1 (FMRIB Software Library) as part of a larger study on the effects of exercise activity on white matter microstructure in children (Chaddock-Heyman et al., 2018). Preprocessing of each participant’s data consisted of (1) motion and eddy current correction, (2) removal of non-brain tissue using the Brain Extraction Tool (Smith, 2002), and (3) local fitting of the diffusion tensor model at each voxel using FMRIB’s Diffusion Toolbox v2.0 (FDT). These steps yielded fractional anisotropy (FA) and first, second, and third eigenvalue (L1, L2, L3) maps. The first eigenvalue map was used as the axial diffusivity (AD) image, whereas the mean of the second and third eigenvalue maps was used as the radial diffusivity (RD) image (Song et al., 2002).

Having obtained whole-brain FA, AD, and RD images, tract-based diffusion maps were defined using TBSS v1.2 [Tract-Based Spatial Statistics (Smith et al., 2006)]. Each participant’s FA map was aligned into the 1 mm × 1 mm × 1 mm standard Montreal Neurological Institute (MNI152) space *via* the FMRIB58_FA template using the FMRIB’s non-linear registration tool (Andersson et al., 2007a,b), and a mean diffusion image was created. The mean FA image was then thinned to create an average skeleton representing the centers of the tracts shared by all participants, and the skeleton was thresholded at FA > 0.20. Each participant’s aligned FA data were projected onto the skeleton to obtain FA skeleton values for each individual. RD and AD skeletons for each participant were formed in a similar manner by projecting the RD and AD maps onto the mean skeleton.

Tract ROIs were created from the JHU ICBM-DTI-81 white matter labels atlas (Mori and van Zijl, 2007; Wakana et al., 2007) in the left and right superior longitudinal fasciculus, as well as the genu, body, and splenium of the corpus callosum. Diffusion values (FA, RD, AD) were calculated for each participant within each of the tract ROIs. FA, AD, and RD values of each tract ROI were exported to SPSS for analysis.

### Statistical Analyses

Our primary behavioral hypothesis was that children who played a musical instrument would show higher scores on verbal ability and intellectual ability as assessed by the Woodcock-Johnson tests of cognitive abilities, even after accounting for the possible effects of age, sex, socio-economic status, and fitness. This was assessed using ANCOVAs comparing the standardized scores on verbal ability and intellectual ability between children who do and do not play a musical instrument, while incorporating age, sex SES, and VO_2_max as covariates.

Our primary neuroimaging hypothesis was that children who played a musical instrument would show differences in microstructural properties of the SLF and CC, as indexed by FA, AD, and RD in these regions, compared to children who did not play a musical instrument. This was assessed using a multivariate general linear model (GLM) with the FA, AD, and RD of left and right SLF and the genu, body, and splenium of the corpus callosum as dependent variables, playing an instrument as a fixed factor, and age, sex, SES, and aerobic fitness (VO_2_max) as covariates.

Our secondary hypothesis was that the same microstructural properties identified above would correlate with the intensity of musical practice. This was assessed by partial correlations between the number of hours of music practice per week and diffusion parameters that showed significant between-subject effects in the multivariate analysis above, with age, sex, SES, and VO_2_max as covariates.

## Results

### Behavioral Results

Participants who were reported as participating in musical activities (*n* = 47) performed significantly higher than their counterparts (*n* = 53) on verbal ability (with musical activities: mean = 114.6, SD = 10.05; without musical activities: mean = 106.00, SD = 12.38) and intellectual ability (with musical activities: mean = 114.77, SD = 11.56; without musical activities: mean = 107.16, SD = 11.97). A one-way ANCOVA on the dependent variable of verbal ability, with the independent variables of musical activities and the covariates of age, sex, SES, and VO_2_max, showed a significant effect of musical activities [*F*(1,94) = 12.56, *p* = 0.001, partial *η*^2^ = 0.12] and no significant effect of age, sex, SES, and VO_2_max [age: *F*(1,94) = 0.083, *p* = 0.77, partial *η*^2^ = 0.001; sex: *F*(1,92) = 0.46, *p* = 0.50, partial *η*^2^ = 0.005; SES: *F*(1,94) = 0.036, *p* = 0.85, partial *η*^2^ < 0.001; VO_2_max: *F*(1,94) = 3.72, *p* = 0.057, partial *η*^2^ = 0.12]. A one-way ANCOVA on the dependent variable of intellectual ability, with the independent variables of musical activities and the same covariates, showed a significant effect of musical activities (*F*(1,94) = 7.42, *p* = 0.008, partial *η*^2^ = 0.075), while age sex, SES, and VO_2_max were all not significant [age: *F*(1,94) = 1.89, *p* = 0.17, partial *η*^2^ = 0.020; sex: *F*(1,94) = 2.09, *p* = 0.15, partial *η*^2^ = 0.022; SES: *F*(1,94) = 0.51, *p* = 0.48, partial *η*^2^ = 0.005; VO_2_max: *F*(1,94) = 3.43, *p* = 0.067, partial *η*^2^ = 0.036].

**Figure 1:**
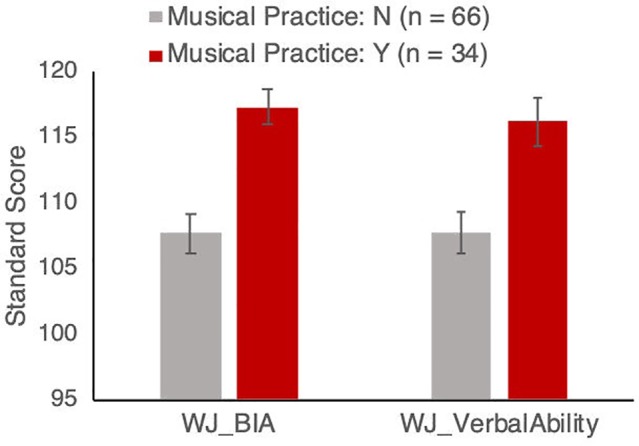
Woodcock-Johnson Brief Intellectual Ability (BIA) and Verbal Ability standardized scores for groups with and without musical instrument training. Results remain significant after controlling for age, sex, socio-economic status, and aerobic fitness, as described in the text. Error bars show between-subject standard error of the mean.

Within the group that were reported as participating in musical activities, those who were reported to play a musical instrument (*n* = 34) had a range of 0.5–6 h of practice per week, with a mean of 1.275 (SD = 1.434) h of practice per week. The instruments they play included piano (*n* = 20), violin (*n* = 5), guitar (*n* = 5), recorder (*n* = 3), and drums (*n* = 2). Participants who play a musical instrument scored higher on verbal ability compared to those who reported not playing a musical instrument (instrument players mean = 116, SD = 10.67; non-players mean = 107.63, SD = 11.44; see Figure 1). This was confirmed with a one-way ANCOVA comparing standard scores of verbal ability between children who do and do not report playing a musical instrument, with age, sex, SES, and VO_2_max as covariates: the effect of playing an instrument was highly significant [*F*(1, 94) = 9.10, *p* = 0.003, partial *η*^2^ = 0.091]. The covariates of age, sex, SES, and VO_2_max were all not significant [age: *F*(1,94) = 0.721, *p* = 0.40, partial *η*^2^ = 0.008; sex: *F*(1,94) = 0.59, *p* = 0.44, partial *η*^2^ = 0.006; SES: *F*(1,94) = 0.09, *p* = 0.76, partial *η*^2^ = 0.001; VO_2_max: *F*(1,94) = 1.58, *p* = 0.21, partial *η*^2^ = 0.017].

Participants who play a musical instrument also scored higher on the overall Brief Intellectual Ability test (instrument players mean = 117.27, SD = 9.37; non-players mean = 107.69, SD = 12.46; Figure 1). This was again confirmed with an ANCOVA comparing children who do and do not report playing a musical instrument, with age, sex, SES, and VO_2_max as covariates: the effect of playing an instrument was highly significant (*F*(1, 94) = 14.88, *p* < 0.001, partial *η*^2^ = 0.138). The covariates of age, sex, SES, and VO_2_max were not significant [age: *F*(1,94) = 4.648, *p* = 0.033, partial *η*^2^ = 0.033; sex: *F*(1,94) = 1.23, *p* = 0.27, partial *η*^2^ = 0.009; SES: *F*(1,94) = 1.019, *p* = 0.315, partial *η*^2^ = 0.007; VO_2_max: *F*(1,94) = 3.01, *p* = 0.085, partial *η*^2^ = 0.022].

In contrast to playing a musical instrument, participants who report singing in choir (*n* = 15) did not score higher on verbal ability or on intellectual ability than those who did not report singing in choir (*n* = 85) (intellectual ability: choir mean = 110.13, SD = 13.68; non-choir mean = 110.64, SD = 12.13. Verbal ability: choir mean = 110.33, SD = 10.675; non-choir mean = 109.90, SD = 11.781; all *p* > 0.2; all partial *η* ^2^ < 0.05).

We further examined the relationship between the intensity of musical practice, quantified as number of hours of reported music practice per week, and verbal ability and intellectual ability in a partial correlation controlling for age, sex, SES, and VO_2_max. Practice intensity was significantly correlated with verbal ability (*r_p_* = 0.260, *p_p_* = 0.041), but not significantly correlated with intellectual ability (*r_p_* = 0.21, *p_p_* = 0.099).

### Neuroimaging Results

**Figure 2:**
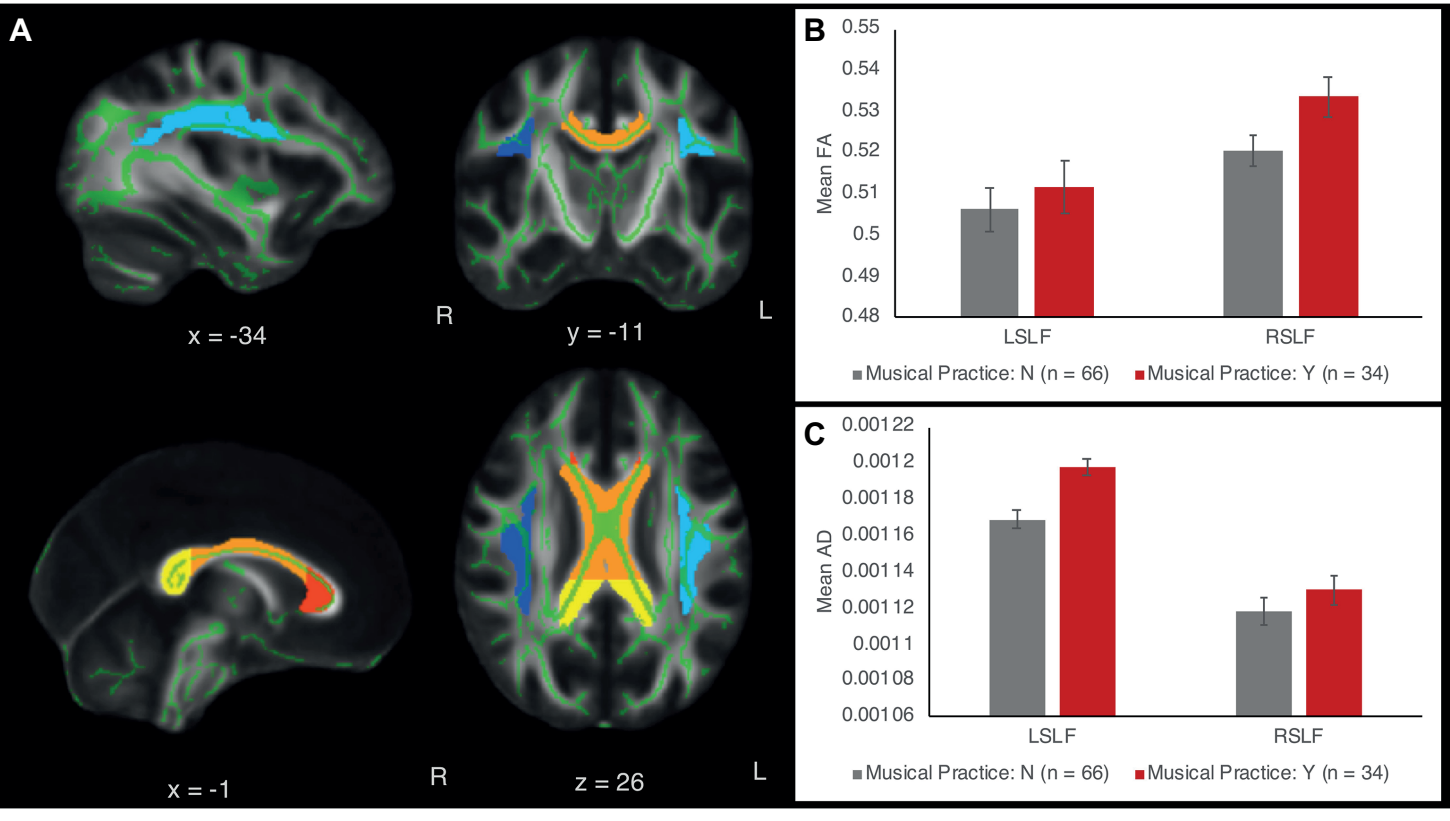
**(A)** White matter tract ROIs in the left and right superior longitudinal fasciculus (SLF) and the corpus callosum (CC). Light blue = left SLF; dark blue = right SLF; red = genu of CC; orange = body of CC; yellow = splenium of CC. The ROIs are overlaid on the standard white matter skeleton (green) and 1 mm template FA image (grayscale). Green voxels inside the ROIs are averaged across the ROI to obtain mean FA, AD, and RD values across the ROI. **(B)** FA of the left and right SLF as a function of musical expertise. **(C)** AD of left and right SLF as a function of musical expertise. Error bars show between-subject standard error of the mean.

Since the behavioral results showed that whether or not children played a musical instrument was most predictive of verbal and intellectual abilities, we first compared children who did and did not play a musical instrument in a multivariate ANCOVA with all the diffusion measures (FA, AD, and RD) in the left and right SLF and CC (genus, body, splenium) as outcomes variables, while controlling for age, sex, SES, and VO_2_max, the same four covariates as in behavioral analyses. The effect of playing a musical instrument on diffusion measures (considering FA, AD, and RD together) was highly significant [*F*(15,75) = 3.84, *p* < 0.001, partial *η*^2^ = 0.44] (Figure 2). The covariate of age on the diffusion measures was also significant [*F*(15,75) = 2.059, *p* = 0.022, partial *η*^2^ = 0.29]. The effects of SES and VO_2_max were not significant at the 0.05 level [SES: *F*(15,75) = 1.64, *p* = 0.083, partial *η*^2^ = 0.25; VO_2_max: *F*(15,75) = 1.06, *p* = 0.40, partial *η*^2^ = 0.18).

Follow-up univariate ANCOVAs were conducted to test for between-subject differences in each diffusion measure for each tract ROI. Since we were testing three types of diffusion measures (FA, AD, and RD) and five tract ROIs (right SLF, left SLF, CC genu, CC body, and CC splenium), Bonferroni correction at the *p* < 0.05 level was applied for 3 × 5 = 15 statistical tests. The results showed that instrument players had higher AD values in the left SLF [*F*(1,91) = 15.64, *p* < 0.001, partial *η* ^2^ = 0.149], surviving Bonferroni correction at the *p* < 0.05 level. No other FA, AD, or RD values in the left or right SLF were significant at the Bonferroni-corrected level. FA, AD, and RD values in the corpus callosum were similar between children with and without musical instrument training (all *p*’s >0.1). Table 2 shows means and standard deviations of each diffusion measure for musically trained and untrained participants.

**Table 2:** Means and standard deviations of diffusion parameters for participants with and without musical instrument training in each region of interest in the superior longitudinal fasciculus (SLF) and corpus callosum (CC).

			Musical instrument training			
			No (*n* = 66)		Yes (*n* = 34)	
Tract	Diffusion statistic	ROI	Mean	SD	Mean	SD
SLF	FA	SLF right	0.521	0.031	0.534	0.028
		SLF left	0.506	0.044	0.512	0.037
	AD	SLF right	1.12E-03	3.51E-05	1.13E-03	4.68E-05
		SLF left^*^	1.17E-03	3.90E-05	1.20E-03	4.22E-05
	RD	SLF right	4.77E-04	3.42E-05	4.68E-04	2.98E-05
		SLF left	5.32E-04	3.64E-05	5.22E-04	2.35E-05
CC	FA	CC genu	0.752	0.022	0.755	0.017
		CC body	0.698	0.033	0.697	0.027
		CC splenium	0.794	0.019	0.792	0.021
	AD	CC genu	1.48E-03	5.42E-05	1.49E-03	5.03E-05
		CC body	1.54E-03	4.58E-05	1.55E-03	6.39E-05
		CC splenium	1.56E-03	4.75E-05	1.57E-03	9.85E-05
	RD	CC genu	3.17E-04	3.09E-05	3.11E-04	2.37E-05
		CC body	3.94E-04	4.68E-05	3.98E-04	4.03E-05
		CC splenium	2.76E-04	2.81E-05	2.81E-04	3.47E-05

Effects of musical instrument training, controlling for covariates of age, sex, SES, and fitness: *p < 0.001, surviving Bonferroni correction at *p* < 0.05 level for 15 comparisons.

The covariate of age was also significant in FA of the right SLF [*F*(1,91) = 11.562, *p* = 0.001, partial *η*^2^ = 0.115] and in RD of the left and right SLF [left SLF: *F*(1,91) = 8.956, *p* = 0.004, partial *η*^2^ = 0.091; right SLF: *F*(1,91) = 6.557, *p* = 0.012, partial *η*^2^ = 0.069]. Older children had higher FA and lower RD, consistent with previous reports (Krogsrud et al., 2016).

Using participation in music activities (instead of playing a musical instrument) as a predictor in the multivariate test yielded the same significant effect of participation in musical activities [*F*(15,75) = 2.58, *p* = 0.004, partial *η* ^2^ = 0.34], but the covariates of age, sex, SES, and VO_2_max were not significant (all *p* > 0.1). Follow-up ANCOVA again showed that AD in the left SLF was significantly higher among children who participated in music activities [*F*(1,91) = 8.955, *p* = 0.004, partial *η*^2^ = 0.090]; this was significant at the *p* < 0.05 level but not at the Bonferroni-corrected level. Substituting singing in choir as a predictor in the same multivariate analysis yielded no significant effect of choir singing [*F*(15, 73) = 0.79, *p* = 0.68, partial *η*^2^ = 0.14) and no significant covariates (all *p*’s > 0.1).

We further examined the relationship between the intensity of musical practice and FA, AD, and RD in the left and right SLF. FA and RD were not significantly correlated with practice intensity; however, a significant positive correlation was found between practice intensity and axial diffusivity (AD) in the left SLF (Spearman rank-order correlation *r_s_* = 0.305, *p_s_* = 0.011). The association between AD and practice intensity remained significant after controlling for differences in age, sex, socioeconomic status, and VO_2_max (partial correlation: *r_p_* = 0.319, *p_p_* = 0.012). AD of the left SLF was also correlated with intellectual ability (Spearman rank-order correlation *r_s_* = 0.165, *p_s_* = 0.04) but not significantly with verbal ability (Spearman rank-order correlation *r_s_* = 0.140, *p_s_* = 0.095).

## Discussion

Here, we show that children who participate in musical activities, specifically by playing one or more musical instruments for at least 0.5 h per week, have higher verbal ability and better general intellectual ability, as assessed by standardized neuropsychological measures of cognitive performance. Furthermore, music training and cognitive outcome variables are associated with measures of white matter in the superior longitudinal fasciculus, a major white matter pathway in the brain. Effects are significant even after controlling for differences in age, sex, socio-economic status, and aerobic fitness. As early to middle childhood are periods of rapid cognitive and brain development, participation in cultural and/or artistic activities during these periods may have lasting effects on brain and cognitive health.

The present study relates music training and cognitive performance to white matter structure in a large population of children. In this sample, music training also predicted intellectual ability, corresponding to previous findings from randomized controlled trials on music learning (Moreno et al., 2011). The behavioral results are consistent with a meta-analysis on effects of musical training on literacy skills, which found support for the hypothesis that music training leads to gains in phonological awareness skills, albeit with a small effect size relative to the large variance in these skills across different ages and different family backgrounds (Gordon et al., 2015). Recent work has shown that children who have musical training also possess enhanced phonological processing abilities, with fMRI results showing more left-lateralized temporoparietal activity during phonological processing in musically trained children (Zuk et al., 2018). Our findings are broadly consistent with these results as we show that while right SLF is sensitive to differences between children with and without musical instrumental training, these differences could also be explained by age and SES; in contrast, AD of the left SLF is sensitive to training and practice intensity.

Diffusion properties of white matter in the SLF has been related to musical training and musical pitch identification abilities (Oechslin et al., 2010; Halwani et al., 2011; Moore et al., 2017), as well as to the ability to learn language (Floel et al., 2009; Qi et al., 2015) and music (Loui et al., 2011; Vaquero et al., 2018). FA in the left SLF predicts reading ability as defined by phonological awareness tests in childhood (Yeatman et al., 2011; Saygin et al., 2013); the same variable is also positively correlated with language exposure in childhood as defined by conversational turns in children’s environment (Romeo et al., 2018). In the right hemisphere, FA in the right SLF is positively correlated with Mandarin Chinese learning success (Qi et al., 2015) and with pitch-related grammar learning (Loui et al., 2011). Here, we show that while FA and RD are related to age, AD of the left SLF is most strongly correlated with the intensity of musical practice. One limitation is that the resolution of the diffusion images is relatively low (3.44 mm × 3.44 mm × 4 mm), which could affect useful indices such as FA (Barrio-Arranz et al., 2015). FA measures anisotropy of the diffusion tensor and is useful as a general index of white matter as it is sensitive to several properties such as myelination, axonal diameter, and coherence of axonal fibers (but see Jones et al., 2013). RD is the average of the second and third eigenvalues of the diffusion tensor and may be related to myelination (Song et al., 2002); however, cautious interpretations are necessary due to issues with crossing fibers (Wheeler-Kingshott and Cercignani, 2009). In contrast, AD describes water mobility along the axis of the main fiber orientation (Jones et al., 2013). While RD increase has been linked to demyelination in animal models (Song et al., 2002), changes in AD tend to be more variable. Decreases in AD have been observed in axonal injury in animal models, specifically in mice inflicted with retinal ischemia, which in the early stage shows axonal damage without myelin damage (Song et al., 2003; Sun et al., 2006). Budde et al. (2009) also linked AD decrease to axonal damage, rather than myelination, in the mouse spinal cord. While these animal studies link axonal injury to AD decrease, AD of white matter tracts has been reported to increase during brain maturation (Alexander et al., 2011); specifically AD in the SLF has been shown to increase with age during adolescence (Ashtari et al., 2007). However, other studies have observed decreases, or little to no changes, in AD throughout the course of development (Lebel et al., 2008, 2012; Lebel and Beaulieu, 2011; Simmonds et al., 2014; Krogsrud et al., 2016; Moura et al., 2016; Seunarine et al., 2016). Based on these findings, one possible interpretation is that the observed increases in AD among children who practice musical instruments reflect greater coherence (movement along the same direction) of the mobility of water molecules along the principal direction of orientation of the SLF, likely linked to increased maturation or coherence of axons; this maturation or coherence is also associated with the intensity of musical practice. This is qualified by the fact that the relatively low spatial resolution in the present imaging parameters may limit our interpretations due to possible partial voluming. Future studies (e.g., using newer sequences and more detailed image analysis) will further disentangle the diffusion properties that contribute to white matter during brain development.

Our current results are consistent with recent work showing that a perisylvian network is changed by music training (Habibi et al., 2017; Zuk et al., 2018). Interestingly, and contrary to recent findings (Steele et al., 2013; Habibi et al., 2017), our results did not show musical training-related differences in corpus callosum specifically as a result of musical training. Part of this difference may arise from the fact that the present sample involves children from a restricted age; furthermore, we did not collect data on the age of onset of musical training; thus, we could not compare early and late starting musicians as in other reports (Steele et al., 2013). Moreover, recent results from the same population found that children who engage in a 9-month physical activity program had changes in FA and RD of the corpus callosum, compared to an untrained (wait-listed) control group (Chaddock-Heyman et al., 2018). As playing a musical instrument is a mild form of physical activity, results from these different lines of work may suggest that any physical activity that increases oxygen consumption may change the corpus callosum, along with a multitude of other changes in brain health (Hillman et al., 2008; Chaddock et al., 2011), whereas playing a musical instrument may more specifically influence auditory-motor regions especially the SLF, with more rigorous forms of musical training extending toward effects in the corpus callosum. Some support for this comes from the observation that children with musical training in this sample have slightly higher VO_2_max (Table 1), indicating slightly higher fitness than their musically untrained counterparts. Although this was not statistically significant in the present sample, future work with a larger sample may specifically investigate the relationship between musical training and aerobic fitness. At present, by incorporating VO_2_max as a covariate in our main analyses relating music training to neuropsychological and neuroimaging measures, we rule out the alternative explanation that the effects of musical training might be explained by aerobic fitness.

Interestingly, participation in choir did not predict cognitive measures in this sample. This may be because only a small subset (15%) of our sample participated in choir. Although previous reports have shown positive effects of music and singing on wellbeing in adults (Daykin et al., 2017), there remains a need for research on the cognitive effects of singing training with younger populations (Demorest and Pfordresher, 2015). Also unlike previous reports (Oechslin et al., 2010), we did not observe a reversal in hemispheric asymmetry of the SLF in musicians. This difference may arise from differences in DTI methodology: while other reports simultaneously investigated volume (number of voxels) and FA of the SLF (Oechslin et al., 2010; Halwani et al., 2011), we used the tract-based spatial statistics approach, which aligns all participants’ white matter skeletons such that the number of voxels within each ROI is the same across participants; this allows for a robust comparison of diffusion statistics (FA, AD, RD) in the same voxels across participants. Continued investigation with the current dataset may involve probabilistic tractography to trace the SLF over individually defined ROIs and to enable more methodologically similar comparisons with previous reports.

In the current study, we limit our neuroimaging analyses to white matter microstructural properties of SLF and CC, as they are regions for which we have *a priori* hypotheses from previous literature on white matter effects of musical training. Future work may additionally consider the effects of musical instrumental practice on other white matter pathways, as well as on other brain measures such as resting state or task-related fMRI and EEG/ERP indices of executive function and other cognitive processes.

## Conclusion

The results suggest that the relationship between musical practice and intellectual ability is related to axonal fibers in white matter pathways in the auditory-motor system.

## Ethics Statement

This study was carried out in accordance with the recommendations of IRB of UIUC with written informed consent from all subjects. All subjects gave written informed consent in accordance with the Declaration of Helsinki. The protocol was approved by the Institutional Review Board at the University of Illinois. Parents provided written informed consent, and participants provided written assent.

## Author Contributions

PL conceptualized the idea behind this manuscript, performed data analyses, and wrote the first draft. LR, LC-H, AK, and CH conceptualized and designed the larger study, for which the data were obtained. LR and LC-H acquired and preprocessed the behavioral and neuroimaging data. All authors revised the manuscript and approved the submission.

### Conflict of Interest Statement

The authors declare that the research was conducted in the absence of any commercial or financial relationships that could be construed as a potential conflict of interest.

## Footnotes

^1^Free or reduced lunch is a national meal program that operates in public and nonprofit private schools. Eligibility is based on the national poverty line: students whose family income is at or below 130% of the poverty line are eligible for free lunch; students whose family income is at or below 180% of the poverty line are eligible for reduced price lunch.

## References

[ref1] Alexander A. L. Hurley S. A. Samsonov A. A. Adluru N. Hosseinbor A. P. Mossahebi P.. (2011). Characterization of cerebral white matter properties using quantitative magnetic resonance imaging stains. Brain Connect. 1, 423–446. 10.1089/brain.2011.0071, PMID: 22432902 PMC3360545

[ref2] Andersson J. L. Jenkinson M. Smith S. (2007a). Non-linear optimisation. FMRIB technical report TR07JA1. (Oxford, UK: University of Oxford FMRIB Centre).

[ref3] Andersson J. L. Jenkinson M. Smith S. (2007b). Non-linear registration, aka spatial normalisation FMRIB technical report TR07JA2. FMRIB Analysis Group of the University of Oxford 2.

[ref4] Anvari S. H. Trainor L. J. Woodside J. Levy B. A. (2002). Relations among musical skills, phonological processing, and early reading ability in preschool children. J. Exp. Child Psychol. 83, 111–130. 10.1016/S0022-0965(02)00124-8, PMID: 12408958

[ref5] Ashtari M. Cervellione K. L. Hasan K. M. Wu J. McIlree C. Kester H.. (2007). White matter development during late adolescence in healthy males: a cross-sectional diffusion tensor imaging study. NeuroImage 35, 501–510. 10.1016/j.neuroimage.2006.10.047, PMID: 17258911

[ref6] Bailey J. A. Zatorre R. J. Penhune V. B. (2014). Early musical training is linked to gray matter structure in the ventral premotor cortex and auditory-motor rhythm synchronization performance. J. Cogn. Neurosci. 26, 755–767. 10.1162/jocn_a_00527, PMID: 24236696

[ref7] Bar-Or O. (Ed.) (1983). “Physiologic responses to exercise of the healthy child” in Pediatric sports medicine for the practitioner. (New York, NY: Springer), 1–65.

[ref8] Barrio-Arranz G. de Luis-García R. Tristán-Vega A. Martín-Fernández M. Aja-Fernández S. (2015). Impact of MR acquisition parameters on DTI scalar indexes: a tractography based approach. PLoS One 10:e0137905. 10.1371/journal.pone.0137905, PMID: 26457415 PMC4601730

[ref9] Bermudez P. Lerch J. P. Evans A. C. Zatorre R. J. (2009). Neuroanatomical correlates of musicianship as revealed by cortical thickness and voxel-based morphometry. Cereb. Cortex 19, 1583–1596. 10.1093/cercor/bhn196, PMID: 19073623

[ref10] Budde M. D. Xie M. Cross A. H. Song S.-K. (2009). Axial diffusivity is the primary correlate of axonal injury in the experimental autoimmune encephalomyelitis spinal cord: a quantitative pixelwise analysis. J. Neurosci. 29, 2805–2813. 10.1523/JNEUROSCI.4605-08.2009, PMID: 19261876 PMC2673458

[ref11] Chaddock L. Neider M. B. Lutz A. Hillman C. H. Kramer A. F. (2011). Role of childhood aerobic fitness in successful street crossing. Med. Sci. Sports Exerc. 44, 749–753. 10.1249/MSS.0b013e31823a90cb, PMID: 21986808

[ref12] Chaddock-Heyman L. Erickson K. I. Holtrop J. L. Voss M. W. Pontifex M. B. Raine L. B.. (2014). Aerobic fitness is associated with greater white matter integrity in children. Front. Hum. Neurosci. 8:584. 10.3389/fnhum.2014.00584, PMID: 25191243 PMC4137385

[ref13] Chaddock-Heyman L. Erickson K. I. Kienzler C. Drollette E. S. Raine L. B. Kao S.-C. (2018). Physical activity increases white matter microstructure in children. Front. Neurosci. 12:950. 30618578 10.3389/fnins.2018.00950 PMC6305717

[ref14] Daykin N. Mansfield L. Meads C. Julier G. Tomlinson A. Payne A. (2017). What works for wellbeing? A systematic review of wellbeing outcomes for music and singing in adults. Perspect. Public Health 138, 39–46. 29130840 10.1177/1757913917740391 PMC5753835

[ref16] Demorest S. M. Pfordresher P. Q. (2015). Singing accuracy development from k-adult: a comparative study. Music. Percept. 32, 293–302. 10.1525/mp.2015.32.3.293

[ref17] Donnelly J. E. Hillman C. H. Castelli D. Etnier J. L. Lee S. Tomporowski P.. (2016). Physical activity, fitness, cognitive function, and academic achievement in children: a systematic review. Med. Sci. Sports Exerc. 48, 1197–1222. 10.1249/MSS.0000000000000901, PMID: 27182986 PMC4874515

[ref18] Ellis R. J. Bruijn B. Norton A. C. Winner E. Schlaug G. (2013). Training-mediated leftward asymmetries during music processing: a cross-sectional and longitudinal fMRI analysis. NeuroImage 75, 97–107. 10.1016/j.neuroimage.2013.02.045, PMID: 23470982 PMC3705762

[ref19] Floel A. de Vries M. H. Scholz J. Breitenstein C. Johansen-Berg H. (2009). White matter integrity in the vicinity of Broca’s area predicts grammar learning success. NeuroImage 47, 1974–1981. 10.1016/j.neuroimage.2009.05.046, PMID: 19477281

[ref20] Freedson P. S. Goodman T. L. (1993). “Measurement of oxygen consumption” in Pediatric laboratory exercise testing: clinical guidelines, ed. Rowland T. W., (Champaign, Il: Human Kinestics), 91–113.

[ref21] Gardiner M. F. Fox A. Knowles F. Jeffrey D. (1996). Learning improved by arts training. Nature 381:284. 10.1038/381284a0, PMID: 8692266

[ref22] Gordon R. L. Fehd H. M. McCandliss B. D. (2015). Does music training enhance literacy skills? A meta-analysis. Front. Psychol. 6:1777. 10.3389/fpsyg.2015.01777, PMID: 26648880 PMC4664655

[ref23] Gullick M. M. Demir-Lira O. E. Booth J. R. (2016). Reading skill-fractional anisotropy relationships in visuospatial tracts diverge depending on socioeconomic status. Dev. Sci. 19, 673–685. 10.1111/desc.12428, PMID: 27412229 PMC5995108

[ref24] Habibi A. Damasio A. Ilari B. Veiga R. Joshi A. A. Leahy R. M. (2017). Childhood music training induces change in micro and macroscopic brain structure: results from a longitudinal study. Cereb. Cortex 28, 4336–4347. 10.1093/cercor/bhx286 29126181

[ref25] Halwani G. F. Loui P. Rueber T. Schlaug G. (2011). Effects of practice and experience on the arcuate fasciculus: comparing singers, instrumentalists, and non-musicians. Front. Psychol. 2. 10.3389/fpsyg.2011.00156, PMID: 21779271 PMC3133864

[ref26] Hillman C. H. Erickson K. I. Kramer A. F. (2008). Be smart, exercise your heart: exercise effects on brain and cognition. Nat. Rev. Neurosci. 9, 58–65. 10.1038/nrn2298, PMID: 18094706

[ref27] Hillman C. H. Pontifex M. B. Castelli D. M. Khan N. A. Raine L. B. Scudder M. R.. (2014). Effects of the FITKids randomized controlled trial on executive control and brain function. Pediatrics 134:e1063. 10.1542/peds.2013-3219, PMID: 25266425 PMC4179093

[ref28] Ho Y. C. Cheung M. C. Chan A. S. (2003). Music training improves verbal but not visual memory: cross-sectional and longitudinal explorations in children. Neuropsychology 17, 439–450. 10.1037/0894-4105.17.3.439, PMID: 12959510

[ref29] Hurwitz I. Wolff P. H. Bortnick B. D. Kokas K. (1975). Nonmusical effects of the Kodaly music curriculum in primary grade children. J. Learn. Disabil. 8, 167–174.

[ref30] Hyde K. L. Lerch J. Norton A. Forgeard M. Winner E. Evans A. C.. (2009). Musical training shapes structural brain development. J. Neurosci. 29, 3019–3025. 10.1523/JNEUROSCI.5118-08.2009, PMID: 19279238 PMC2996392

[ref31] Jaschke A. C. Honing H. Scherder E. J. A. (2018). Longitudinal analysis of music education on executive functions in primary school children. Front. Neurosci. 12:103. 29541017 10.3389/fnins.2018.00103 PMC5835523

[ref32] Jones D. K. Knosche T. R. Turner R. (2013). White matter integrity, fiber count, and other fallacies: the do’s and don’ts of diffusion MRI. NeuroImage 73, 239–254. 10.1016/j.neuroimage.2012.06.081, PMID: 22846632

[ref33] Karpati F. J. Giacosa C. Foster N. E. V. Penhune V. B. Hyde K. L. (2017). Dance and music share gray matter structural correlates. Brain Res. 1657, 62–73. 10.1016/j.brainres.2016.11.029, PMID: 27923638

[ref34] Khan N. A. Hillman C. H. (2014). The relation of childhood physical activity and aerobic fitness to brain function and cognition: a review. Pediatr. Exerc. Sci. 26, 138–146. 10.1123/pes.2013-0125, PMID: 24722921

[ref35] Kramer A. F. Hahn S. Cohen N. J. Banich M. T. McAuley E. Harrison C. R.. (1999). Ageing, fitness and neurocognitive function. Nature 400, 418–419. 10.1038/22682, PMID: 10440369

[ref36] Kraus N. Chandrasekaran B. (2010). Music training for the development of auditory skills. Nat. Rev. Neurosci. 11, 599–605. 10.1038/nrn2882, PMID: 20648064

[ref37] Krogsrud S. K. Fjell A. M. Tamnes C. K. Grydeland H. Mork L. Due-Tønnessen P.. (2016). Changes in white matter microstructure in the developing brain—a longitudinal diffusion tensor imaging study of children from 4 to 11years of age. NeuroImage 124, 473–486. 10.1016/j.neuroimage.2015.09.017, PMID: 26375208 PMC4655940

[ref38] Lamb S. J. Gregory A. H. (1993). The relationship between music and reading in beginning readers. Educ. Psychol. 13, 19–27. 10.1080/0144341930130103

[ref39] Lebel C. Beaulieu C. (2011). Longitudinal development of human brain wiring continues from childhood into adulthood. J. Neurosci. 31, 10937–10947. 10.1523/JNEUROSCI.5302-10.2011, PMID: 21795544 PMC6623097

[ref40] Lebel C. Gee M. Camicioli R. Wieler M. Martin W. Beaulieu C. (2012). Diffusion tensor imaging of white matter tract evolution over the lifespan. NeuroImage 60, 340–352. 10.1016/j.neuroimage.2011.11.094, PMID: 22178809

[ref41] Lebel C. Walker L. Leemans A. Phillips L. Beaulieu C. (2008). Microstructural maturation of the human brain from childhood to adulthood. NeuroImage 40, 1044–1055. 10.1016/j.neuroimage.2007.12.053, PMID: 18295509

[ref42] Loui P. Alsop D. Schlaug G. (2009). Tone deafness: a new disconnection syndrome? J. Neurosci. 29, 10215–10220. 10.1523/JNEUROSCI.1701-09.2009, PMID: 19692596 PMC2747525

[ref43] Loui P. Li H. C. Schlaug G. (2011). White matter integrity in right hemisphere predicts pitch-related grammar learning. NeuroImage 55, 500–507. 10.1016/j.neuroimage.2010.12.022, PMID: 21168517 PMC3035724

[ref44] Lynn R. Wilson R. G. Gault A. (1989). Simple musical tests as measures of Spearman’s g. Vol. 10 (Netherlands: Elsevier Science), 25–28.

[ref45] Mehr S. A. Schachner A. Katz R. C. Spelke E. S. (2013). Two randomized trials provide no consistent evidence for nonmusical cognitive benefits of brief preschool music enrichment. PLoS One 8:e82007. 10.1371/journal.pone.0082007, PMID: 24349171 PMC3859544

[ref46] Moore E. Schaefer R. S. Bastin M. E. Roberts N. Overy K. (2017). Diffusion tensor MRI tractography reveals increased fractional anisotropy (FA) in arcuate fasciculus following music-cued motor training. Brain Cogn. 116, 40–46. 10.1016/j.bandc.2017.05.001, PMID: 28618361 PMC5479403

[ref47] Moreno S. Bialystok E. Barac R. Schellenberg E. G. Cepeda N. J. Chau T. (2011). Short-term music training enhances verbal intelligence and executive function. Psychol. Sci. 22, 1425–1433. 10.1177/0956797611416999, PMID: 21969312 PMC3449320

[ref48] Mori S. van Zijl P. (2007). Human white matter atlas. Am. J. Psychiatry 164:1005. 10.1176/ajp.2007.164.7.1005, PMID: 17606649

[ref49] Moura L. M. Kempton M. Barker G. Salum G. Gadelha A. Pan P. M.. (2016). Age-effects in white matter using associated diffusion tensor imaging and magnetization transfer ratio during late childhood and early adolescence. Magn. Reson. Imaging 34, 529–534. 10.1016/j.mri.2015.12.021, PMID: 26708037

[ref50] Oechslin M. S. Imfeld A. Loenneker T. Meyer M. Jancke L. (2010). The plasticity of the superior longitudinal fasciculus as a function of musical expertise: a diffusion tensor imaging study. Front. Hum. Neurosci. 3, 1–12. 10.3389/neuro.09.076.2009 PMC2821183 20161812

[ref51] Qi Z. Han M. Garel K. San Chen E. Gabrieli J. D. E. (2015). White-matter structure in the right hemisphere predicts mandarin Chinese learning success. J. Neurolinguistics 33, 14–28. 10.1016/j.jneuroling.2014.08.004

[ref52] Roden I. Kreutz G. Bongard S. (2012). Effects of a school-based instrumental music program on verbal and visual memory in primary school children: a longitudinal study. Front. Neurosci. 6:572. 10.3389/fpsyg.2012.00572 PMC3528082 23267341

[ref53] Romeo R. R. Segaran J. Leonard J. A. Robinson S. T. West M. R. Mackey A. P.. (2018). Language exposure relates to structural neural connectivity in childhood. J. Neurosci. 38, 7870–7877. 10.1523/JNEUROSCI.0484-18.2018, PMID: 30104336 PMC6125810

[ref54] Sachs M. Kaplan J. Der Sarkissian A. Habibi A. (2017). Increased engagement of the cognitive control network associated with music training in children during an fMRI Stroop task. PLoS One 12:e0187254. 10.1371/journal.pone.0187254, PMID: 29084283 PMC5662181

[ref55] Sala G. Gobet F. (2017). Does far transfer exist? Negative evidence from chess, music, and working memory training. Curr. Dir. Psychol. Sci. 26, 515–520. 10.1177/0963721417712760, PMID: 29276344 PMC5724589

[ref56] Saygin Z. M. Norton E. S. Osher D. E. Beach S. D. Cyr A. B. Ozernov-Palchik O.. (2013). Tracking the roots of reading ability: white matter volume and integrity correlate with phonological awareness in prereading and early-reading kindergarten children. J. Neurosci. 33, 13251–13258. 10.1523/JNEUROSCI.4383-12.2013, PMID: 23946384 PMC3742917

[ref57] Schellenberg E. G. (2004). Music lessons enhance IQ. Psychol. Sci. 15, 511–514. 15270994 10.1111/j.0956-7976.2004.00711.x

[ref58] Schellenberg E. G. (2006). Long-term positive associations between music lessons and IQ. J. Educ. Psychol. 98, 457–468. 10.1037/0022-0663.98.2.457

[ref59] Schlaug G. Jancke L. Huang Y. Staiger J. F. Steinmetz H. (1995). Increased corpus callosum size in musicians. Neuropsychologia 33, 1047–1055. 10.1016/0028-3932(95)00045-5, PMID: 8524453

[ref60] Schrank F. A. (2011). “Woodcock-Johnson III tests of cognitive abilities” in Handbook of pediatric neuropsychology. ed. Davis A. S. (New York, NY, US: Springer Publishing Co.), 415–434.

[ref61] Seunarine K. K. Clayden J. D. Jentschke S. Munoz M. Cooper J. M. Chadwick M. J.. (2016). Sexual dimorphism in white matter developmental trajectories using tract-based spatial statistics. Brain Connect. 6, 37–47. 10.1089/brain.2015.0340, PMID: 26446207 PMC4744889

[ref62] Sihvonen A. J. Ripollés P. Leo V. Rodríguez-Fornells A. Soinila S. Särkämö T. (2016). Neural basis of acquired amusia and its recovery after stroke. J. Neurosci. 36, 8872–8881. 10.1523/JNEUROSCI.0709-16.2016, PMID: 27559169 PMC6601900

[ref63] Sihvonen A. J. Ripolles P. Sarkamo T. Leo V. Rodriguez-Fornells A. Saunavaara J.. (2017). Tracting the neural basis of music: deficient structural connectivity underlying acquired amusia. Cortex 97, 255–273. 10.1016/j.cortex.2017.09.028, PMID: 29100660

[ref64] Simmonds D. J. Hallquist M. N. Asato M. Luna B. (2014). Developmental stages and sex differences of white matter and behavioral development through adolescence: a longitudinal diffusion tensor imaging (DTI) study. NeuroImage 92, 356–368. 10.1016/j.neuroimage.2013.12.044, PMID: 24384150 PMC4301413

[ref65] Sluming V. Barrick T. Howard M. Cezayirli E. Mayes A. Roberts N. (2002). Voxel-based morphometry reveals increased gray matter density in Broca’s area in male symphony orchestra musicians. NeuroImage 17, 1613–1622. 10.1006/nimg.2002.1288, PMID: 12414299

[ref66] Smith S. M. (2002). Fast robust automated brain extraction. Hum. Brain Mapp. 17, 143–155. 10.1002/hbm.10062, PMID: 12391568 PMC6871816

[ref67] Smith S. M. Jenkinson M. Johansen-Berg H. Rueckert D. Nichols T. E. Mackay C. E.. (2006). Tract-based spatial statistics: voxelwise analysis of multi-subject diffusion data. NeuroImage 31, 1487–1505. 10.1016/j.neuroimage.2006.02.024, PMID: 16624579

[ref68] Song S. K. Sun S. W. Ju W. K. Lin S. J. Cross A. H. Neufeld A. H. (2003). Diffusion tensor imaging detects and differentiates axon and myelin degeneration in mouse optic nerve after retinal ischemia. NeuroImage 20, 1714–1722. 10.1016/j.neuroimage.2003.07.005, PMID: 14642481

[ref69] Song S. K. Sun S. W. Ramsbottom M. J. Chang C. Russell J. Cross A. H. (2002). Dysmyelination revealed through MRI as increased radial (but unchanged axial) diffusion of water. NeuroImage 17, 1429–1436. 10.1006/nimg.2002.1267, PMID: 12414282

[ref70] Steele C. J. Bailey J. A. Zatorre R. J. Penhune V. B. (2013). Early musical training and white-matter plasticity in the corpus callosum: evidence for a sensitive period. J. Neurosci. 33, 1282–1290. 10.1523/JNEUROSCI.3578-12.2013, PMID: 23325263 PMC6704889

[ref71] Sun S. W. Liang H. F. Le T. Q. Armstrong R. C. Cross A. H. Song S. K. (2006). Differential sensitivity of in vivo and ex vivo diffusion tensor imaging to evolving optic nerve injury in mice with retinal ischemia. NeuroImage 32, 1195–1204. 10.1016/j.neuroimage.2006.04.212, PMID: 16797189

[ref72] Utter A. C. Robertson R. J. Nieman D. C. Kang J. (2002). Children’s OMNI scale of perceived exertion: walking/running evaluation. Med. Sci. Sports Exerc. 34, 139–144. 10.1097/00005768-200201000-00021, PMID: 11782659

[ref73] Vaquero L. Ramos-Escobar N. Francois C. Penhune V. Rodriguez-Fornells A. (2018). White-matter structural connectivity predicts short-term melody and rhythm learning in non-musicians. NeuroImage. 181, 252–262. 10.1016/j.neuroimage.2018.06.054, PMID: 29929006

[ref74] Wakana S. Caprihan A. Panzenboeck M. M. Fallon J. H. Perry M. Gollub R. L.. (2007). Reproducibility of quantitative tractography methods applied to cerebral white matter. NeuroImage 36, 630–644. 10.1016/j.neuroimage.2007.02.049, PMID: 17481925 PMC2350213

[ref75] Wheeler-Kingshott C. A. Cercignani M. (2009). About “axial” and “radial” diffusivities. Magn. Reson. Med. 61, 1255–1260. 10.1002/mrm.21965, PMID: 19253405

[ref76] Woodcock R. W. Mather N. McGrew K. S. Wendling B. J. (2001). Woodcock-Johnson III tests of cognitive abilities. (Itasca, IL: Riverside Publishing Company).

[ref77] Yeatman J. D. Dougherty R. F. Rykhlevskaia E. Sherbondy A. J. Deutsch G. K. Wandell B. A.. (2011). Anatomical properties of the arcuate fasciculus predict phonological and reading skills in children. J. Cogn. Neurosci. 23, 3304–3317. 10.1162/jocn_a_00061, PMID: 21568636 PMC3214008

[ref78] Zuk J. Benjamin C. Kenyon A. Gaab N. (2014). Behavioral and neural correlates of executive functioning in musicians and non-musicians. PLoS One 9:e99868. 10.1371/journal.pone.0099868, PMID: 24937544 PMC4061064

[ref79] Zuk J. Perdue M. V. Becker B. Yu X. Chang M. Raschle N. M.. (2018). Neural correlates of phonological processing: disrupted in children with dyslexia and enhanced in musically trained children. Dev. Cogn. Neurosci. 34, 82–91. 10.1016/j.dcn.2018.07.001, PMID: 30103188 PMC6481189

